# Sufentanil in Intensive Care: A Narrative Review

**DOI:** 10.3390/jcm15145684

**Published:** 2026-07-20

**Authors:** Jose M. Gomez, Paloma Navarro, Álvaro Mingote-Lladó, Pablo Cardinal-Fernández

**Affiliations:** 1 Hospital General Universitario Gregorio Marañón, 28007 Madrid, Spain; 2 School of Medicine, Universidad Camilo José Celá, 28692 Madrid, Spain; 3 School of Medicine, CEU San Pablo University, 28668 Madrid, Spain; 4 Altan Pharmaceuticals, 28231 Madrid, Spain; 5 Intensive Care Unit, Torrelodones University Hospital, 28250 Madrid, Spain

**Keywords:** sufentanil, analgosedation, intensive care, mechanical ventilation, opioid analgesics, pharmacokinetics, critical care

## Abstract

**Background**: Modern intensive care paradigms prioritize optimal analgesia over heavy sedation. While morphine, fentanyl, and remifentanil are widely used, sufentanil is relatively unknown in several countries. Aimed primarily at intensive care physicians, this narrative review evaluates the utilization of sufentanil in critically ill adult patients across four fundamental dimensions: (1) translational pharmacology (chemical structure, pharmacokinetics, pharmacodynamics, and pharmacogenomics); (2) indications and safety precautions; (3) clinical evidence in ICU patients; and (4) therapeutic positioning in contemporary ICU analgosedation and future research directions. **Methods**: A comprehensive literature search was conducted in PubMed/MEDLINE focusing on the translational pharmacology, safety parameters, and clinical studies of sufentanil critically ill adults. The synthesis was structured according to SANRA principles for high-quality narrative reviews and includes articles published up to February 2026. **Results**: Sufentanil exhibits an exceptionally high affinity for the µ-opioid receptor, providing a potency 10-fold greater than fentanyl and 1000-fold greater than morphine. It has an experimental therapeutic index (LD50/ED50) nearly two orders of magnitude wider than that of fentanyl, enabling safe, highly granular bedside titration. Unlike morphine, hepatic CYP3A4 biotransformation yields completely inactive metabolites, preventing toxic accumulation during acute kidney injury. Furthermore, receptor-binding dynamics suggest a lower propensity for recruiting β-arrestins and activating NMDA pathways, potentially reducing opioid-induced hyperalgesia (OIH). Clinically, pilot trials in neurocritical cohorts demonstrate that sufentanil maintains reliable cerebral hemodynamic stability. In postoperative cardiac surgery and large ICU registries, sufentanil may reduce the mechanical ventilation duration, accelerate extubation, and shorten ICU length of stay. **Conclusions**: For critically ill adults, sufentanil is an excellent alternative to traditional opioids because it offers high-precision titratability, potent analgesia, and a safer metabolic profile.

## 1. Introduction

The critically ill patient is characterized by the actual or potential instability of their major physiological systems; the interventions required for monitoring and controlling these situations routinely generate pain, which negatively impacts the patient’s well-being and prognosis [1]. In 2016, Vincent et al. [2] coined the eCASH (early Comfort using Analgesia, minimal Sedatives and maximal Humane care) concept with the aim of prioritizing analgesia over hypnosis. Subsequently, Marra et al. [3] designed the ABCDEF bundle (Assess, prevent, and manage pain; Both spontaneous awakening and spontaneous breathing trials; Choice of analgesia and sedation; Delirium and Early exercise and mobility, Family engagement and empowerment), which includes pain control as one of its core pillars. Its implementation has been shown to reduce seven major adverse events related to intensive care: hospital mortality, days of mechanical ventilation, coma, delirium, use of physical restraints, intensive care unit (ICU) readmission, and discharge to facilities other than home [4]. For critically ill patients experiencing severe pain, the American PADIS guidelines recommend the use of opioids [5]. The ideal opioid should provide potent analgesia, rapid titratability, hemodynamic stability, low risk of histamine release, predictable offset after prolonged infusion, limited active metabolite accumulation, and compatibility with sedative strategies designed to maintain light sedation. Current recommendations of the working group of the Spanish Society of Intensive Care Medicine and Coronary Units (SEMICYUC) position remifentanil and fentanyl as the first-line agents of choice [6]. Although sufentanil shares certain characteristics with fentanyl and remifentanil, it has a distinct pharmacological profile that makes it unique. In Spain, despite being available for over twelve years, sufentanil remains underutilized in intensive care—a stark contrast to its widespread adoption across other European nations. For instance, a study by Martin et al. [7] in German intensive care units (ICUs) revealed that sufentanil was the preferred opioid in 35% of sedations lasting less than 24 h, 53% of those lasting 24 to 72 h, and 44% of sedations exceeding 72 h. Similarly, the Société de Réanimation de Langue Française (SRLF) identified sufentanil as the primary analgesic in 48% of French ICUs [8]. This discrepancy between high regional adoption and localized underutilization highlights a critical clinical divide. It reflects not only institutional availability and local tradition, but also differences in clinician training, hospital formulary design, and a pervasive perception that fentanyl and remifentanil already satisfy all intensive care analgesic requirements. Such heterogeneity has arguably delayed a balanced re-evaluation of continuous-infusion sufentanil in mechanically ventilated adults. Consequently, a modern review is warranted to align sufentanil’s pharmacology with contemporary ICU liberation paradigms and to identify the specific clinical scenarios that stand to benefit most from its application.

Aimed primarily at intensive care physicians, this narrative review evaluates the utilization of sufentanil in the critical care setting across four fundamental dimensions: (1) translational pharmacology (chemical structure, pharmacokinetics, pharmacodynamics, and pharmacogenomics); (2) indications and safety precautions; (3) clinical evidence in ICU patients; and (4) therapeutic positioning in contemporary ICU analgosedation and future research directions.

## 2. Methods

This article was developed as a structured narrative review. The objective was to synthesize the pharmacological rationale and clinical evidence supporting the use of sufentanil in adult intensive care. A targeted literature search was conducted in PubMed/MEDLINE and major guideline sources using combinations of the terms sufentanil, intensive care, critical care, mechanical ventilation, sedation, analgesia, analgosedation, fentanyl, remifentanil, morphine, opioid-induced hyperalgesia, pharmacokinetics, pharmacodynamics, pharmacogenomics, extracorporeal membrane oxygenation (ECMO), renal replacement therapy, and delirium. Regulatory product information and society guideline documents were also reviewed when dosing, contraindications, or safety warnings were discussed.

As a narrative review, this work utilizes a selective synthesis of literature focusing on adult ICU populations or reporting ICU-specific outcomes (e.g., length of stay or mortality) rather than quantitative pooling; consequently, formal PRISMA flow reporting and meta-analytic methods were not applied. To ensure methodological rigor, the narrative structure was guided by the Scale for the Assessment of Narrative Review Articles (SANRA) guidelines. The literature search included articles published up to February 2026. In accordance with the SANRA benchmarks, this review maintains a clear clinical aim, an explicit selection process, appropriate referencing, sound scientific reasoning, and a balanced interpretation of the evidence [9].

## 3. Results

### 3.1. Translational Pharmacology

In 1959, Janssen Pharmaceutica synthesized a new phenylpiperidine-derived molecule named fentanyl. From this base, several synthetic opioids were subsequently developed, including sufentanil, whose defining characteristic is its exceptionally high therapeutic potency—10 times greater than fentanyl and 1000 times greater than morphine [10,11,12].

The molecular structure of sufentanil consists of a central piperidine ring substituted with an anilidophenylpropionamide core, a methoxymethyl group at the 4-position, and a thienylethyl chain. While the piperidine scaffold and phenylpropionamide group are shared with other fentanyl derivatives, the thienyl moiety and methoxymethyl substitution are unique modifications that drastically increase the drug’s lipid solubility and µ-opioid receptor affinity. This specific chemical profile translates directly into rapid equilibration across the blood–brain barrier after intravenous administration, high analgesic potency at low plasma concentrations, and a negligible tendency toward histamine-mediated vasodilation compared with morphine.

Under physiological conditions, its pharmacokinetics follow a three-compartment distribution model, with a mean distribution half-life to peripheral tissues of 1.4 min and a redistribution half-life of 17 min. The analgesic effect is maintained for 30 to 50 min. This drug is highly lipophilic, enabling rapid passage across the blood–brain barrier. Its high ionization (80% at physiological pH with a pKa of 8.0) and high plasma protein binding (93.0%)—predominantly to albumin and alpha-1-acid glycoprotein—result in a relatively small volume of distribution, which is smaller than that of fentanyl (Table 1) [13,14].

The termination of the sufentanil clinical effect depends primarily on drug redistribution, a substantial difference compared with remifentanil, whose termination depends entirely on plasma and tissue esterases. Sufentanil excretion occurs predominantly via metabolism through the cytochrome P450 enzyme complex, specifically CYP3A4, into inactive metabolites (mainly N-phenyl-propanamide). This feature constitutes a major difference from morphine, whose active metabolite (morphine-6-glucuronide) prolongs secondary adverse effects. The inactive metabolites of sufentanil are eliminated by the kidneys, while a small fraction of sufentanil and its metabolites is excreted via bile and feces [15].

Critical illness can modify each component of this pharmacokinetic profile. Hypoalbuminemia and changes in alpha-1-acid glycoprotein alter the relationship between total and unbound drug concentration. Shock, vasopressor exposure, hypothermia, systemic inflammation, and reduced hepatic perfusion can modify distribution and clearance. In prolonged infusions, the clinically relevant variable is not only the terminal half-life, but also the context-sensitive half-time, which determines the time required for plasma concentrations to fall after stopping the infusion. This concept is essential when sufentanil is compared with both fentanyl and remifentanil. Remifentanil has the most stable offset because of organ-independent esterase metabolism; however, sufentanil also has a clinically short context-sensitive half-time after intermediate-duration infusions and remains substantially more predictable than fentanyl, whose context-sensitive half-time increases markedly with infusion duration. This distinction is relevant in ICU patients who require sustained analgesia, but in whom delayed awakening and ventilator liberation are undesirable [15,16,17,18].

Advanced organ support techniques may also modify sufentanil’s pharmacokinetics. For instance, Hahn et al. [19] analyzed 20 critically ill patients on ECMO who received sufentanil-based analgesia and sedation. They described substantial deviations from standard physiological pharmacokinetics, specifically reporting a two-compartment distribution model with first-order elimination, an increased volume of distribution (Vd), and decreased clearance [20,21].

In patients with severe renal impairment, while morphine carries the risk of accumulating its active metabolite, sufentanil utilizes an alternative elimination pathway, thus offering a critical advantage in conditions such as septic shock, postoperative illness, and multiorgan failure, where acute kidney injury is common. Conversely, navigating hepatic impairment requires a completely different clinical approach. Because sufentanil relies heavily on extensive intestinal and hepatic biotransformation via the CYP3A4 pathway, its clearance is highly vulnerable to fluctuations in liver blood flow, intrinsic enzyme activity, protein binding, and competing drug interactions. As a result, conditions such as severe liver failure, low-flow shock, hypothermia, or the co-administration of potent CYP3A4 inhibitors (e.g., azole antifungals, macrolide antibiotics, amiodarone, etc.) can significantly prolong drug exposure. Managing these high-risk scenarios demands proactive dose reductions and more gradual titration, coupled with vigilant monitoring for delayed awakening, respiratory depression, bradycardia, and hypotension [15,22].

From a pharmacodynamic standpoint, like other opioids, sufentanil acts on µ, δ, and κ receptors, which are widely distributed throughout the central (spinal cord and brain) and peripheral nervous systems. The binding capacity of an opioid to its receptor is described by the affinity constant Ki (where a lower Ki value denotes higher affinity), which correlates directly with pharmacological potency [23]. Clinically, opioid-receptor binding translates to an increased pain threshold, altered conscious pain perception and emotional response, and inhibition of both ascending pain pathways and the associated sympathetic response. Sufentanil exhibits a higher affinity for the µ receptor (a lower Ki) than fentanyl and remifentanil. This means that it achieves the same analgesic and sedative effects with fewer molecules, thereby minimizing potential administration-related adverse effects [24].

Opioid-induced hyperalgesia (OIH) is characterized by a state of nociceptive sensitization triggered by opioid administration [25,26]. While tolerance necessitates higher dosing to achieve an equivalent analgesic effect, OIH presents a distinct clinical challenge: increasing the dose paradoxically exacerbates pain [27]. In the intensive care unit, the clinical impact of OIH extends far beyond elevated pain scores. Because critically ill patients frequently receive prolonged opioid infusions and undergo repetitive painful procedures, they are particularly vulnerable. Consequently, OIH can manifest as allodynia, agitation, severe sleep disruption, or a prolonged, complicated opioid weaning process, often compounded by withdrawal symptoms. Some studies suggest that sufentanil may induce OIH less frequently than related drugs such as remifentanil, which represents a substantial clinical advantage over other opioids [28]. This unexpected behavior could be linked to its higher affinity for µ receptors, lower activation of NMDA receptors, and lower production of pronociceptive metabolites [29,30,31]. Additionally, sufentanil may trigger lower recruitment of β-arrestins—proteins responsible for the desensitization and internalization of µ receptors, as well as alternative signaling pathways linked to OIH, tolerance, and adverse effects such as respiratory depression and nausea [16,32].

From a pharmacogenomic perspective, several polymorphisms have been linked to sufentanil metabolism. One of the most studied is CYP3A4 (rs2242480), which involves a guanine-to-adenosine substitution; while it does not alter the protein structure, it results in reduced cytochrome function, and consequently, lower sufentanil dose requirements [33,34,35]. The OPRM1 A118G (rs1799971) polymorphism substitutes adenine for guanine in the mu-opioid receptor, altering opioid affinity. Some studies associate the G allele with lower receptor activity and the need for higher clinical doses of sufentanil [36]. Finally, the ABCB1 3435 (rs1045642) variant, which substitutes cytosine for thymine without affecting the amino acid sequence, can modulate P-glycoprotein expression at the blood–brain barrier, thereby altering the cerebral availability of sufentanil [37].

Although these pharmacogenomic variables explain a significant portion of the interindividual variability observed in opioid responses, routine pre-treatment genetic screening is not currently recommended or logistically feasible in the acute ICU setting. Instead, these genetic insights underscore why fixed dosing strategies often fail in critical care. In daily clinical practice, rather than relying on genomic profiles, clinicians must simply depend on meticulous, effect-driven titration guided by validated objective tools—such as pain behavioral scales and sedation scores—to safely navigate these underlying genetic differences at the bedside.

### 3.2. Indications and Safety Precautions

In Spain, sufentanil is marketed for intravenous administration in two distinct presentations: 2 mL ampoules at a concentration of 5 mcg/mL (10 µg per ampoule) and 10 mL ampoules at a concentration of 50 µg/mL (250 µg per ampoule). According to its summary of product characteristics, its approved adult indications encompass prolonged sedation in intensive care or resuscitation settings for mechanically ventilated patients, as well as an analgesic adjuvant for maintaining balanced general anesthesia of medium-to-long duration when combined with hypnotics, volatile agents, and muscle relaxants [22]. Furthermore, it is indicated as a primary anesthetic for the induction and maintenance of analgesic anesthesia with 100% oxygen during major surgical interventions, such as cardiovascular procedures and via the epidural route—either as single or repeated boluses or as a continuous infusion, alone or alongside local anesthetics—for surgical, obstetric, or postoperative management.

When managing prolonged sedation during mechanical ventilation, intravenous sufentanil is recommended as a continuous infusion ranging from 0.2 to 2 µg/kg/h, meticulously tailored to the patient’s analgesic needs and concomitant sedative regimens. To optimize these continuous infusion parameters in non-surgical, mechanically ventilated adult patients, supplementing sufentanil with low-dose esketamine hydrochloride represents a highly effective, multimodal clinical strategy. This combination leverages a powerful pharmacological synergy by pairing the potent µ-opioid receptor agonism of sufentanil with the NMDA receptor antagonism of esketamine, which actively prevents opioid-induced hyperalgesia and acute tolerance. By blocking these secondary pain pathways, esketamine exerts a profound opioid-sparing effect, allowing clinicians to achieve targeted sedation and ventilator synchrony at significantly lower cumulative doses of sufentanil. Furthermore, this dual regimen enhances safety by stabilizing physiological equilibrium; esketamine’s mild sympathetic stimulation counterbalances the dose-dependent bradycardia and hypotension often induced by high-dose opioids, while its direct bronchodilatory effects improve lung compliance in acute medical respiratory pathologies without causing central respiratory depression. Nonetheless, intensivists must remain vigilant regarding esketamine-specific risks, particularly hypersalivation and transient neuropsychiatric manifestations, such as emergence delirium or hallucinations during the weaning phase.

For postoperative pain management, the epidural route offers an elegant alternative, delivered either as intermittent boluses of 0.75 µg/kg/h diluted in a 10-mL volume (typically 25 to 30 µg/kg/h administered at the earliest signs of waning analgesia) or as a continuous epidural infusion maintained between 0.2 and 0.3 µg/kg/h.

Beyond its metabolic pathway, a compelling pharmacological feature of sufentanil is its potential standing as the safest agent in its class with respect to experimental overdose margins. In comparative animal models, the therapeutic index—expressed as the ratio of the median lethal dose to the median effective dose (LD50/ED50)—is approximately 26,700 for sufentanil, exceeding the 277–300 range reported for fentanyl by nearly two orders of magnitude. For the clinician, this large experimental safety profile must not be mistaken for absolute, unsupervised safety; as a full µ-opioid receptor agonist, sufentanil can still precipitate dose-dependent respiratory depression, bradycardia, hypotension, and chest wall rigidity without careful monitoring. Instead, the true bedside implication of this vast therapeutic window is its profound impact on precision titratability. Within a structured ICU protocol, this unmatched separation between the effective analgesic dose and lethal toxicity allows intensivists to execute highly granular microgram-scale adjustments. This exceptional margin of safety helps explain why sufentanil maintains a remarkably stable hemodynamic and respiratory profile, even during rapid acute titration in critically ill patients [11,38,39].

Despite this favorable safety margin, the main contraindications of sufentanil must be strictly respected [22]. These include hypersensitivity to the active substance, its excipients, or other morphine derivatives, as well as the concomitant use of monoamine oxidase inhibitors (MAOIs) or mixed morphine agonists/antagonists such as nalbuphine, buprenorphine, and pentazocine. It is important to highlight that nalbuphine, an infrequently used analgesic in Spain, competitively blocks the µ-receptor; therefore, it is fundamentally incompatible with opioid-based analgesic regimens. Its co-administration would abruptly reverse the potent analgesic effects of sufentanil, triggering severe breakthrough pain or acute withdrawal at the bedside. Additionally, sufentanil is contraindicated in any coexisting treatments or conditions that preclude epidural administration, such as severe hemorrhage, shock, systemic sepsis, injection site infections, hemostatic disorders, or ongoing anticoagulant therapy.

While the broader safety precautions for using sufentanil in critically ill adults are generally shared with the wider opioid class, the drug possesses several noteworthy clinical considerations [22]. Deep analgesia is frequently accompanied by marked respiratory depression; however, in a study of 30 healthy volunteers, Bailey et al. [38] reported that the onset of respiratory depression is slower and recovery is faster with sufentanil than with fentanyl, despite sufentanil having a longer duration of analgesia. Cardiovascular depression and hypotension may also occur, particularly in patients with preexisting hypovolemia, when an inadequate dose of anticholinergics has been administered, or when sufentanil is combined with non-vagolytic muscle relaxants such as rocuronium, vecuronium, or cisatracurium. Furthermore, clinicians must remain vigilant regarding acute chest wall rigidity and transient elevations in intracranial pressure (ICP). Consequently, proactive dose reductions are recommended in elderly or debilitated patients, individuals classified as ASA III/IV, or those with uncontrolled hypothyroidism, chronic alcohol abuse, pulmonary disease, or hepatic and renal impairment.

In practical terms, a sound implementation approach is to initiate therapy at the lower end of the recommended infusion range in elderly, frail, hypovolemic, vasopressor-dependent patients or patients with hepatic impairment, as well as those targeted for deep sedation. Titration should then proceed dynamically based on pain scores, ventilator synchrony, hemodynamic responses, and established wakefulness targets. Finally, during liberation from mechanical ventilation, the sufentanil infusion should be decreased in parallel with sedation down-titration, unless ongoing severe pain, postoperative status, trauma, burns, or the presence of invasive devices justify maintaining a higher analgesic intensity.

### 3.3. Clinical Evidence in ICU Patients

The clinical evidence can be grouped into two main domains: (a) randomized controlled trials (RCTs) and (b) observational studies.

*a. Randomized controlled trials*

In the neurocritical population, effective analgesia is foundational to stability, as maintaining adequate cerebral perfusion pressure, controlling intracranial pressure, and avoiding secondary brain injury are central safety concerns directly influenced by sympathetic stress responses. Bourgoin et al. [40] compared the effects of sedation using ketamine-midazolam versus sufentanil-midazolam in adult neurocritical patients. The duration of mechanical ventilation and ICU length of stay tended to be shorter in the sufentanil group, with no significant differences observed in cerebral hemodynamics (ICP, CPP, and therapy intensity) during the first 4 days in the ICU.

Baillard et al. [41] compared remifentanil-midazolam versus sufentanil-midazolam in patients requiring invasive mechanical ventilation for more than 48 h. Although no differences were found in the overall duration of mechanical ventilation or ICU stay, they reported that the time to weaning from mechanical ventilation was significantly shorter in the remifentanil group and no tolerance to sufentanil was observed.

*b. Observational studies*

The immediate postoperative period of cardiac surgery typically takes place in the ICU, and outcomes are tightly linked to intraoperative interventions. Deshpande et al. [42] compared sufentanil versus fentanyl during intubation in patients aged 15 to 50 years undergoing valvular heart surgery or congenital disease repairs (both drugs administered alongside muscle relaxants and hypnotics). In the operating room, significantly more patients from the sufentanil group could be successfully extubated. Furthermore, among patients requiring postoperative mechanical ventilation, the time on ventilation in minutes was significantly shorter with sufentanil. Although this was a cardiac anesthesia study rather than a primary ICU trial, it is relevant because early extubation and postoperative ventilation time are core fast-track cardiac ICU outcomes. The higher operating-room extubation rate and shorter postoperative ventilation among those requiring ventilation support a potential role for sufentanil in perioperative pathways designed to reduce residual opioid-sedative burden while preserving hemodynamic stability.

In coronary artery bypass graft (CABG) surgery, Butterworth et al. [43] reported that patients who received sufentanil exclusively as their analgesic required 1.9 fewer hours of mechanical ventilation (95% CI: 0.04 to 4.1 h) compared to those receiving only fentanyl. In line with the previous study, but in patients with traumatic brain injury, Xia et al. [44] also reported a significant reduction in both hours of mechanical ventilation and ICU length of stay in comparison with fentanyl.

Wang et al. [45] used a competing-risk model to compare patients who received at least one day of fentanyl, sufentanil, or remifentanil. Compared to fentanyl, sufentanil was associated with a shorter time to extubation and a shorter time to ICU discharge. Notably, these results remained unchanged when stratified by surgical versus nonsurgical status.

Conversely, Futier et al. [46] evaluated the clinical impact of replacing an ICU analgesia protocol based on remifentanil with one based on sufentanil. The research group reported that remifentanil administration was associated with a reduction in mechanical ventilation duration; however, when stratifying by short-term (less than 4 days) versus prolonged (more than 4 days) mechanical ventilation, this difference was found to be driven entirely by the short-term cohort. The total days of ICU stay were also shorter in the remifentanil group.

**Table 1:** Main pharmacokinetic characteristics of sufentanil, remifentanil, fentanyl, and morphine.

Parameter/Characteristic	Sufentanil	Remifentanil	Fentanyl	Morphine
Class	μ receptor agonist	μ receptor agonist	μ receptor agonist	μ, δ, and κ-opioid receptor agonist
Potency vs. morphine *	Approximately 1000-fold	Approximately 200–300-fold	Approximately 100-fold	Reference
Onset of action, IV *	<1 min	1–2 min	<1 min	5–10 min
Duration of action, IV *	20–45 min	5–10 min during continuous infusion	30–60 min	3–4 h
Terminal half-life *	Approximately 2.5–3 h	Approximately 9–10 min	Approximately 3–4 h	Approximately 2–3 h
Context-sensitive half-time *	30–35 min after 4 h infusion; increases after very prolonged infusion	Approximately constant, 3–4 min	Approximately 180–260 min after 4 h infusion; increases further with prolonged infusion	Not routinely used for titration
Volume of distribution *	Approximately 1.7 L/kg in adults	Approximately 0.3–0.4 L/kg in adults	Approximately 3–6 L/kg in adults	Approximately 3–5 L/kg in adults
Protein binding	Approximately 93%	Approximately 70%	Approximately 86–89%	Approximately 35%
Blood–brain barrier penetration	Rapid, high lipophilicity	Very rapid	Rapid	Slow relative to phenylpiperidines
Metabolism	Hepatic, mainly CYP3A4	Plasma and tissue esterases	Hepatic, mainly CYP3A4	Hepatic glucuronidation to M3G and M6G
Elimination	Inactive metabolites, renal excretion	Inactive metabolites, rapid renal excretion	Inactive metabolites, renal excretion	Active M6G metabolite, renal excretion
Histamine release	Not significant	Not significant	Not significant	Clinically relevant in some patients

Abbreviations: IV, intravenous; min, minutes; L/kg, liter per kilogram; M3G, morphine-3-glucuronide; M6G, morphine-6-glucuronide; CYP3A4, Cytochrome P450 Family 3 Subfamily A Member 4. * Values are approximate and vary with age, critical illness, infusion duration, organ function, and co-administered drugs. Data are derived from pharmacokinetic reviews, ICU pharmacokinetic analyses, and opioid clinical pharmacology data [18,19,20,21,44].

### 3.4. Therapeutic Positioning in Contemporary ICU Analgosedation and Future Research Directions

Current ICU evidence does not support the universal superiority of a single opioid over another for all mechanically ventilated patients; rather, available data suggest that opioid selection should be tailored to the expected duration of mechanical ventilation and the desired recovery profile.

**Figure 1:**
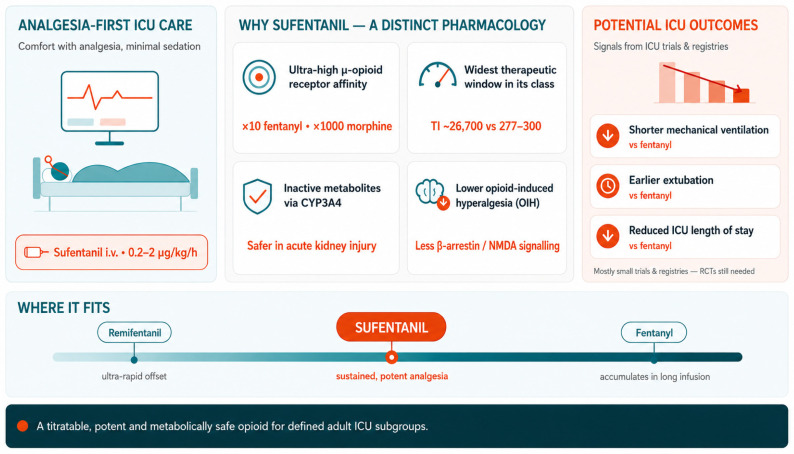
Sufentanil in intensive care. Artificial intelligence-assisted digital art.

In patients with an anticipated short duration of ventilation and a critical need for immediate neurological assessment, remifentanil remains pharmacologically attractive and may be preferable due to its organ-independent, esterase-mediated metabolism and very short context-sensitive half-time. Conversely, for patients requiring longer analgesic coverage, sufentanil offers a distinct pharmacological balance (Figure 1, Table 1 and Table 2). It becomes highly attractive when sustained analgesia is required, particularly in postoperative, trauma, or selected neurocritical populations where rapid onset, high potency, inactive metabolites, a stable hemodynamic profile, the avoidance of morphine metabolite accumulation, and a less abrupt offset are clinically paramount. Supporting this clinical utility, observational data suggest potential reductions in the time to extubation or ICU discharge when using sufentanil compared with fentanyl (Table 3). In contrast, while fentanyl remains familiar and widely available, its lipophilicity, larger volume of distribution, and propensity for accumulation during prolonged infusions can complicate awakening and delay extubation. Similarly, morphine is less suitable as a first-line continuous ICU opioid when renal dysfunction or histamine-mediated hypotension is clinically relevant.

Regarding future research directions, the most important evidence gap is the absence of large, pragmatic, multicenter randomized trials directly comparing sufentanil with fentanyl and remifentanil in adult ICU analgosedation. Future studies should use outcomes that matter to ICU teams and patients: ventilator-free days, time to extubation after readiness criteria are met, ICU and hospital length of stay, delirium/coma-free days, cumulative sedative exposure, opioid withdrawal, opioid-induced hyperalgesia, patient-reported pain and distress after ICU discharge, and mortality.

Trial design should avoid treating all mechanically ventilated patients as a single pharmacological population. Expected ventilation duration, surgical versus medical admission, neurocritical status, shock, renal replacement therapy, ECMO, severe obesity, hepatic dysfunction, and baseline opioid exposure should be prespecified strata. Because sedative choice modifies opioid effects, protocols should standardize or stratify propofol, dexmedetomidine, benzodiazepine exposure, and daily awakening strategies.

Pharmacokinetic-pharmacodynamic substudies are particularly important. They should measure infusion rates, cumulative exposure, organ support variables, protein binding surrogates, sedation and pain scores, respiratory drive during weaning, and recovery after discontinuation. Pharmacogenomic substudies incorporating CYP3A4, OPRM1, and ABCB1 variants could clarify why some patients require higher doses while others experience delayed awakening or adverse effects at apparently standard exposure.

Implementation research is also needed. Countries with high sufentanil utilization may provide valuable real-world data on safety infrastructure, nurse-led titration, pump concentrations, analgesia targets, and extubation workflows. Countries with very low utilization, including Spain and Portugal, offer an opportunity to evaluate structured introduction programs and compare outcomes before and after formulary integration.

## 4. Conclusions

In summary, sufentanil is a synthetic opioid that combines high potency, rapid onset of action, minimal accumulation, and an excellent hemodynamic and safety profile, all of which facilitate effective pain control in the intensive care setting. Remifentanil remains advantageous for the narrow objective of ultra-rapid offset; fentanyl remains a familiar comparator, although available evidence does not demonstrate superior ICU outcomes. Sufentanil occupies a rational intermediate position between these agents and deserves explicit inclusion in comparative ICU analgosedation research.

Moving forward, it will be crucial to directly compare the clinical outcomes of sufentanil versus remifentanil for high-relevance endpoints, such as the duration of mechanical ventilation and ICU length of stay. Future studies should also stratify these effects across specific patient contexts, such as co-administration with different hypnotics or the presence of underlying hepatic impairment.

The next generation of studies should move beyond pharmacological description and evaluate patient-centered and system-level outcomes. Properly designed trials should determine whether sufentanil can reduce ventilator exposure, improve comfort, avoid excessive sedative co-administration, limit opioid-induced hyperalgesia or withdrawal, and support ICU liberation pathways in defined subgroups of critically ill adults.

## Figures and Tables

**Table 2:** Indications and safety precautions of sufentanil in adults critically ill patients.

Category	Clinical Specifications & Parameters
Approved Adult Indications	• Prolonged sedation in intensive care or resuscitation settings for mechanically ventilated patients. • Analgesic adjuvant for maintaining balanced general anesthesia (medium-to-long duration). • Primary anesthetic for induction and maintenance of analgesic anesthesia (with 100% oxygen) in major surgery (e.g., cardiovascular procedures). • Epidural administration (single/repeated bolus or continuous infusion) for surgical, obstetric, or postoperative management.
Absolute Contraindications	• Hypersensitivity to sufentanil, excipients, or other morphine derivatives. • Concomitant use of monoamine oxidase inhibitors or mixed morphine agonists/antagonists (e.g., nalbuphine, buprenorphine, pentazocine). • Epidural-specific contraindications: severe hemorrhage, shock, systemic sepsis, injection site infections, hemostatic disorders, or ongoing anticoagulant therapy.
Recommended Dosing Regimens	• Prolonged sedation (IV continuous infusion): 0.2 to 2 µg/kg/h (tailored to analgesic needs and concurrent sedatives). • Postoperative pain (epidural intermittent bolus): 0.75 µg/kg/h diluted in 10 mL (typically 25 to 30 µg at earliest signs of waning analgesia). • Postoperative pain (epidural continuous infusion): 0.2 to 0.3 µg/kg/h.
Clinical Adjustments & Titration	• Proactive dose reductions: mandatory in elderly, debilitated, American Society of Anesthesiologists (ASA) Physical Status Classification System III/IV, uncontrolled hypothyroidism, chronic alcohol abuse, pulmonary disease, or hepatic/renal impairment. • Initiation strategy: start at the lower end of the infusion range in frail, hypovolemic, vasopressor-dependent, or hepatically impaired patients. • Weaning/liberation: decrease infusion in parallel with sedation down-titration, unless maintained analgesic intensity is justified (e.g., severe pain, trauma, burns, invasive devices).

Abbreviations: µg/kg/h, micrograms per kilogram of body weight per hour; µg, micrograms; mL, milliliters.

**Table 3:** Main studies related to sufentanil in adult critically ill patients.

Author (Year of Publication, Country)	Study Design	Population	Comparators	Key Results (Group A vs. Group B vs. Group C Respectively)
Part 1				
Bourgoin et al. [40] (2003, France)	RCT 1:1, double-blind fashion by using the balanced eight-block sequence generated from a random table.	**Inclusion criteria:** 16–75 yrs who had sustained a TBI resulting in a postresuscitation GCS of 3–8, who required MV and ICP monitoring because of a postresuscitation GCS score < 8, and for whom CT scan results indicated a significant risk of increased ICP. **Exclusion criteria:** life-threatening multiple injuries, kidney or heart insufficiency	Measurements were carried out during the first 4 days of sedation. The average infusion rates during this time were: **group A** (n = 12) 82 ± 25 μg.kg^−1^·min^−1^ ketamine and 1.64 ± 0.5 g·kg^−1^·min^−1^ midazolam; **group B** (n = 13) sufentanil group 0.008 ± 0.002 g.kg^−1^·min^−1^ sufentanil and 1.63 ± 0.37 g·kg^−1^·min^−1^ midazolam.	* Mortality: 4 vs. 3 (*p* ns) * ICU stay (days): 21 ± 13 vs. 18 ± 13 (*p* ns) * Mean duration of sedation (days): 6.1 ± 3.2 vs. 5.3 ± 3.7 (*p* ns) * No significant differences observed in cerebral hemodynamics (ICP, CPP, and therapy intensity) during the first 4 days in the ICU * Mean daily cost (USD/day and patient): 47 ± 13 vs. 42 ± 14 (*p* ns)
Baillard et al. [41] (2004, France)	RCT using opaque envelopes (randomization table), 1:1, double blind. The study was stopped after an interim analysis.	**Inclusion criteria**: IMV with an expected duration of >48 h. **Exclusion criteria**: age < 18, substance abuse, alcoholism, ongoing pregnancy, COPD and neuromuscular disease.	Midazolam was used in both groups as a continuous intravenous infusion at an initial dosage of 0.1 mg/kg per hour. Initial dose: **group A** (n = 21) remifentanil 10 μg/kg per hour (160 μg/mL) and **group B** (n = 20) sufentanil 0.125 μg/kg per hour (2 μg/mL)	* Mortality: 13 and group B 13 (*p* ns) * Median duration of sedation (days): 6 [4; 19] vs. 6 [3; 16] (*p* ns) * Duration of weaning from MV (hours): 22 (12–53) vs. 96 (47–142) (*p* = 0.04) * Withdrawal syndrome (patients): 3 vs. 1 (*p* ns) * Development of tachyphylaxis (patients): 6 after a median sedation duration of 8.5 (6–13) days vs. 0 (*p* = 0.02) * ICU stay (days): 26 (8–45) vs. 19 (11–34) days (*p* ns)
Deshpande et al. [42] (2009, India)	RCT, prospective, double blinded study.	**Inclusion criteria:** age of 15–50 years, undergoing elective open-heart surgery for valvular and simple congenital heart disease. **Exclusion criteria**: excluded patients with LVEF < 20%, severe pulmonary hypertension, severe COPD, renal insufficiency, severe liver disease, history of seizure or stroke, history of allergy to propofol, patients in whom cardiopulmonary bypass time > 2 h and pregnant.	During surgery, **group A** (n = 50): received 0.5 μg.kg^−1^ of sufentanil and **group B** (n = 50) received 3 μg.kg^−1^ of fentanyl as part of induction. All patients were induced with IV midazolam 0.05 mg.kg^−1^, a sleep dose of thiopentone sodium and IV vecuronium 0.1 mg.kg^−1^ to facilitate endotracheal intubation.	* Extubation in operating room: 64% vs. 38% (*p* = 0.009) * Among patients requiring postoperative mechanical ventilation, time on ventilation (minutes): 63.10 ± 99.99 vs. 119.90 ± 126.99 (*p* = 0.015) * Time to 1st dose of tramadol: 43.70 ± 51.1 vs. 70.68 ± 65.538 (*p* = 0. 025) * ICU length of stay (days): 1.16 ± 0.370 vs. 1.14 ± 0.351 (*p* ns)
Part 2				
Butterworth et al. [43] (1998, United States)	Observational, prospective, multicenter	**Inclusion criteria**: adults undergoing to primary coronary artery by-pass graft surgery. **Exclusion criteria**: emergency surgery, “re do”, and those who die peri-operative.	**Group A** sufentanil (n = 196); **group B** Fentanyl (n = 824)	* Duration of intubation (hours): 12.2 (95% CI 10.4; 14.4) vs. 14.2 (95% CI 12.6; 16.0) (*p* = 0.04) * ICU length of stay (hours): 36.3 (95% CI 30.9; 42.7) vs. 36.2 (95% CI 32.1; 40.8) (*p* ns) * Total post-operative length of stayed (days): 5.7 (95% CI 5.5; 6.2) vs. 5.9 (95% CI 5.5; 6.2 (*p* ns)
Xia et al. [44] (2021, China)	Observational, retrospective	**Inclusion criteria**: brain trauma admitted to ICU	**Group A** (n = 42): sufentanil 0.1 μg/kg and then switched to a micro-syringe pump to continuously pump the 0.1 μg/(kg·h); **group B** (n = 43): fentanyl 1.0 μg/kg and then switched to a micro-syringe pump to continuously pump the 1.0 μg/(kg·h). All patients were given propofol.	* MV (hours): 87.5 ± 18.9 vs. 98.6 ± 20.7 (*p* ns) * ICU length of stay (hours): 103.5 ± 57.2 vs. 143.8 ± 96.7 (*p* ns) * Cost of hospitalization ICU (thousand Yuan): 74.3 ± 10.5 vs. 76.1 ± 9.8 (*p* ns)
Wang et al. [45] (2022, China)	Observational, retrospective	**Inclusion criteria**: MV for more than 24 h. **Exclusion criteria**: patients with incomplete information including date of birth, sex, and discharged diagnosis; age < 18 years or admitted to the pediatric ICU; without receiving any opioids during ICU stay and extremely long ICU stay. If patients experienced multiple episodes of MV, only the first episode of MV treatment for more than 24 h was measured.	**Group A**: fentanyl (n = 4778); **group B** sufentanil (n = 4008) and **group C** remifentanil (n = 2233).	* Compared to group A (competitive risk model), group B was associated with an increased hazard for extubation (HR 1.31, 95% CI 1.20–1.41) and ICU discharge (HR 1.63, 95% CI 1.38–1.92). * No significant differences between group A and group B regarding hazards for ventilator mortality (HR 0.95, 95% CI 0.77–1.17) and ICU mortality (HR 1.03, 95% CI 0.84–1.26). * No significant differences between group A and group B were found for ventilator mortality (HR 0.87, 95% CI 0.50–1.49) and ICU mortality (HR 1.25, 95% CI 0.73–2.16). No significant differences were observed between group B and group C regarding hazards for extubation (HR 1.14, 95% CI 0.92–1.41), ventilator mortality (HR 0.91, 95% CI 0.47–1.76), ICU discharge (HR 1.31, 95% CI 0.81–2.14), and ICU mortality (HR 0.66, 95% CI 0.34–1.28).
Futier et al. [46] (2012, France)	Observational, prospective, unicenter	**Inclusion criteria**: adults patients admitted to the ICU who require IMV. **Exclusion criteria**: patients requiring noninvasive ventilation only and those with brain death at or after ICU admission.	**Group A** (n = 794) sufentanil, 5 µg IV bolus followed by a continuous IV infusion of 10 to 25 µg/h; **group B** (n = 750) remifentanil, continuous IV infusion starting at 0.1 µg/kg/min, no boluses) until the pain, ventilator desynchrony, and agitation were controlled.	* ICU mortality (N %): 193 (24.3) vs. 175 (23.4) (*p* ns) * MV duration (days): 14(3–27) vs. 10 (3–21) (*p* < 0.01). In the subgroup analysis, this difference was significant for patients ventilated less than 4 days (*p* = 0.0035) but was non-significant for patients ventilated more than four days (*p* ns). * ICU length of stay (days): 19 (4–26) vs. 16 (3–22) (*p* < 0.01) * Cost of analgesia-based sedation per patient (euros): 254 ± 5 vs. 265 ± 10 (*p* ns)

Abbreviations: RCT, randomized controlled trial; GCS, Glasgow coma scale; CT, computer tomography; ICP, intracranial pressure; ICU, intensive care unit; CPP, cerebral perfusion pressure; USD, United State Dollar; IMV, invasive mechanical ventilation; COPD, chronic obstructive pulmonary disease; IV, intravenous; HR, hazard ratio; CI, confidence interval.

## Data Availability

No new data were created or analyzed in this study. Data sharing is not applicable to this article.
